# Gender-Specific Frequency Distribution of Hepatitis C Virus Genotypes in Punjab province, Pakistan: A Clinically Significant Descriptive Cross-Sectional Study

**DOI:** 10.7759/cureus.17480

**Published:** 2021-08-27

**Authors:** Anam Yousaf, Atif Ghafoor, Noor Fatima, Muhammad Danish

**Affiliations:** 1 Molecular Biology, Pakistan Kidney and Liver Institute and Research Center, Lahore, PAK; 2 Molecular Pathology, Pakistan Kidney and Liver Institute and Research Center, Lahore, PAK; 3 Molecular Biology, Chaudhry Muhammad Akram Teaching & Research Hospital, Lahore, PAK

**Keywords:** hepatitis c (hcv) infection, punjab, hcv genotype, hepatocellular carcinoma (hcc), liver cirrhosis, chronic liver disease (cld)

## Abstract

Background: Hepatitis C virus (HCV) is the major cause of liver cirrhosis, chronic liver disease, and hepatocellular carcinoma. More than 10 million individuals are living with HCV infection in Pakistan. Due to unawareness, very little information is known about HCV genotype occurrence in Punjab, the largest province of Pakistan. Identification of HCV genotype is very important for HCV treatment because different genotypes of HCV respond differently to antiviral therapy.

Objective: The purpose of this research was to determine the distribution frequency of different HCV genotypes in the Punjab province and to demonstrate the distribution pattern of HCV genotypes in different age groups and sexes.

Materials and Methods: In this study, we performed HCV genotyping of 3692 samples collected from different sites of the Punjab province, Pakistan. Among 3692 samples, 1755 (47.5%) were males and 1937 (52.4%) were females.

Results: A total of 3692 samples were subjected to HCV genotyping and 2977 (81%) patients were genotyped successfully, whereas 715 (19%) patients were found to be HCV not detected. Our study demonstrated that among typeable genotypes, 3a constituted 2582 (69.9%) patients followed by 1a (n = 280) 7.5%, 1b (n = 64) 1.7%, 2a (n = 6) 0.16%, genotype 4 (n = 10) 0.27%, 3+4 (n = 2) 0.56%, 1a+2a (n = 11) 0.29%, 1b+2a (n = 1) 0.02%, 1a+1b (n = 1) 0.02%, and 1a+1b+3 (n = 1) 0.02% patients. HCV genotype distribution was evaluated gender wise and in different age groups like 0-12, 13-18, 19-59, and >60 years. All the HCV genotypes were equally distributed among men and women. The most affected age group was 19-59 years as compared to other age groups.

Conclusion: The most frequently distributed HCV genotype in Punjab was found to be genotype 3a followed by genotype 1a, and only 0.94% of infected patients had a mixed genotype infection. Genotype 1a was found to be increasing significantly in the studied population. With these results, it can be assumed that genotype 3a may be replaced by genotype 1a with the passage of time. If this happens, this situation will be challenging in terms of antiviral therapy.

## Introduction

Hepatitis C virus (HCV) infection is the main source of liver impairment, cirrhosis, and hepatocellular carcinoma (HCC). The Centers for Disease Control and Prevention (CDC) and Chiron identified the Hepatitis C virus in 1989. Despite extensive research for the understanding of the virus and disease, HCV infections continued to propagate worldwide [1]. Across the world, around 71 million people have been estimated to have chronic hepatitis C infection leading to hepatocellular carcinoma. The annual incidence rate of HCV infection is over 3 million [2] and the mortality rate is 0.4 million [3]. Pakistan has the second-largest number of HCV infections globally, with 10 million (~5% of the population) infected people [3]. Risk factors that contribute to the high HCV infection rates in Pakistan include unsterile and used needles in healthcare setups, unscreened blood transfusions [4], intravenous drug users, shavings by barbers, and unsafe medical procedures. Lack of awareness among the general population is a crucial factor involved in the viral transmission and spread of disease [5].

HCV is a positive-sense single-stranded RNA genome of 9.6 kb, which belongs to the family *Flaviviridae*. HCV RNA-dependent-RNA-polymerase (NS5B) lacks the proofreading capability, which results in a high level of sequence heterogeneity [6]. HCV is categorized into six genotypes and numerous distinct subtypes based on sequence homology [7]. The percentages of HCV prevalence in all the provinces of Pakistan are as follows: Punjab, 5.46%; Khyber Pakhtunkhwa, 6.07%; Sindh, 2.55%; and Baluchistan, 25.77% [8]. The distribution of HCV genotypes globally is highly varying. Genotypes 1a and 1b have been distributed in the United States and Europe [9], respectively, whereas genotype 2 is constrained to the Middle East and West Africa. Genotype 4 is predominant in countries of Northern and Central Africa, particularly Egypt. Additionally, genotypes 5 and 6 have been found in South Africa and Asia [10]. Several types of research have indicated that genotype 3a has been reported generally in South Asia and Australia [11]. Variations in the relative frequency distributions and epidemiological patterns of several genotypes have been observed in recent years. Current researches have designated an increase in the incidence of genotype 2a, predominantly in Khyber Pakhtunkhwa [12].

Distribution patterns of HCV genotypes in several geographical locations of Pakistan have been studied in different retrospective researches [13]. As the dissemination pattern of HCV genotype has been changed with time, there is a need to reveal it through epidemiological surveys, among the population of Pakistan to enable preventive strategies and alternative treatments [14]. Ramifications of earlier studies conducted in Pakistan are contradictory, and all the researches have relied on specific geographical locations with a limited sample number. We have directed the current study to assume the prevailing pattern of HCV genotypes on a relatively larger scale, including 24 various geographical locations of Pakistan. This will help to assist the therapeutic options and explore the prognostic implications of HCV infection in Pakistan.

## Materials and methods

The study was conducted at PKLI&RC (Pakistan Kidney and Liver Institute and Research Center, Lahore, Pakistan) Laboratory from April 2017 to April 2019. Seropositive patients' blood plasma samples were received from local and peripheral clinics located in 24 different districts of Punjab and performed in Hepatitis Prevention and Treatment Clinic (HPTC) and PKLI. A total of 3692 samples of different age groups like 0-12, 13-18, 19-59, and >60 years were collected for HCV genotyping and stored at -40°C till processing. With the Abbott m2000 (sp/rt) instrument, which was used to process HCV genotype samples, the Abbott RealTime Genotype II assay was performed to determine the different genotypes of HCV in the plasma/serum of HCV-infected individuals.

HCV RNA extraction and detection

Samples were performed in Abbott m2000sp for the extraction of the HCV RNA genome and amplified in Abbott m2000rt for the detection of the genome using the Abbott RealTime HCV Amplification Reagent Kit and Abbott RealTime HCV-GT Control Kit (positive and negative).

HCV genotyping

The Abbott RealTime HCV Genotype II assay is an in vitro reverse transcription-polymerase chain reaction (RT-PCR) assay. It was performed with the Abbott m2000 Sample Preparation System reagents and the Abbott m2000sp and m2000rt instruments for the qualitative identification of HCV genotypes 1(1a, 1b), type-2, type-3, type-4, type-5, and type-6 in plasma or serum from the samples of chronically infected HCV patients.

## Results

In this study, 3692 HCV seropositive patients of different age groups such as 0-12, 13-18, 19-59, and >60 years have maximum viral loads and are subjected to HCV genotyping.

Distribution pattern of hepatitis C virus genotypes in the male and female population studied

**Figure 1:**
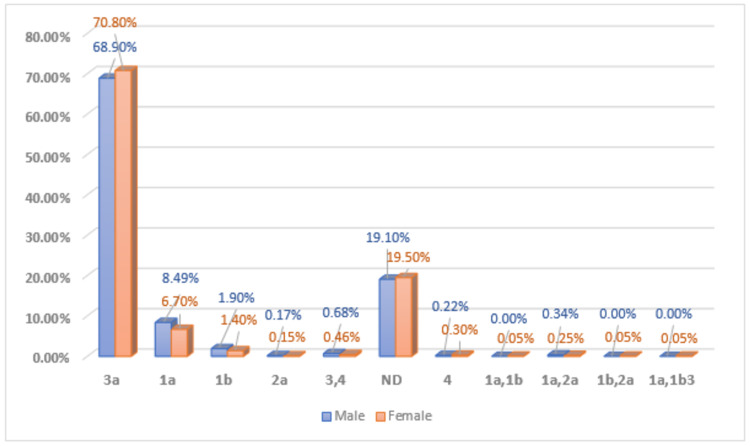
Genderwise distribution of HCV genotypes in the studied population HCV, Hepatitis C virus.

Of the 3692 patients, the prevalence rate in female patients was higher (n = 1937) 52.46% as compared to male patients (n = 1755) 47.54%. Of the total 3692 processed samples, different HCV genotypes were detected in (n = 2977) 81% of patients, whereas (n = 715) 19% of patients were found to be not detected as HCV as the RNA genome was not detected in these patients. Genotype 3 was found to be the most prevalent one among all other HCV genotypes, in this study. It was detected in (n = 1210) 68.90% of male patients, whereas it was detected in (n = 1372) 70.80% of female patients. Prevalence of genotype 1a was (n = 149) 8.49% in male patients and (n = 131) 6.70% in female patients, followed by 1b (n = 35) 1.90% in male patients and (n = 29) 1.40% in female patients. Genotype 2a was found to be detected as (n = 3) 0.17% in male patients and (n = 3) 0.15% in female patients. Genotype 4 was present in (n = 4) 0.22% of male patients and (n = 6) 0.30% of female patients. Mixed genotypes like genotype 3 coinfection with genotype 4 were having the prevalence of (n = 12) 0.68% in male patients and (n = 9) 0.46% in female patients, followed by 1a+2 (n = 6) 0.34% in male patients and (n = 5) 0.25% in female patients. Genotypes in combination like 1a+1b, 1b+2, and 1a+1b+3 were found to be present in (n = 1) 0.05% of female patients (Figure 1).

Age distribution of hepatitis C virus genotypes in infected patients

There were 52 male and 34 female patients in the age group 0-12; 47 male and 25 female patients in the age group 13-18; 1494 male and 1624 female patients in the age group 19-59; and 163 male and 255 female patients in the age group > 60.

**Figure 2:**
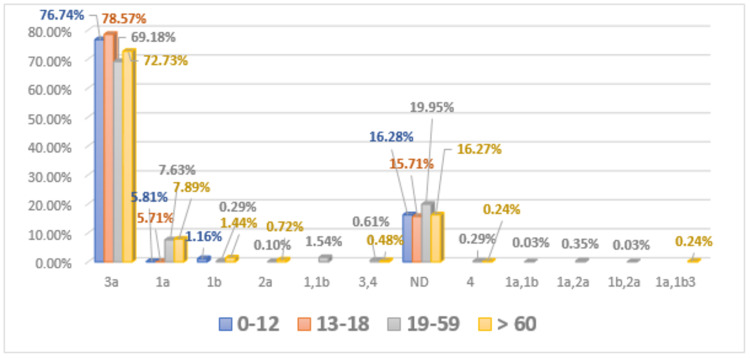
Age-wise distribution of HCV genotypes in the examined population HCV, Hepatitis C virus.

The prevalence of HCV genotypes in patients of various age groups like 0-12, 13-18, 19-59, and >60 is presented in Figure 2. In this study, the prevalence of genotype 3 was found to be (n = 66) 76%, genotype 1a (n = 5) 5.81%, 1b (n = 1) 1.16%, and (n = 14) 16.28% patients were not detected in age group 0-12. Distribution of genotype 3 was determined as (n = 55) 78.57%, genotype 1a (n = 4) 5.71%, and (n = 11) 15.71% patients were not detected in age group 13-18. In age group 19-59, the prevalence of genotype 3 was (n = 2157) 69.18%, 1a (n = 238) 7.63%, 1b (n = 9) 0.29%, 2a (n = 3) 0.10%, genotype 4 (n = 9) 0.29%, mixed genotypes like 1+1b (n = 48) 1.54%, 3+4 (n = 19) 0.61%, 1a+1b (n = 1) 0.03%, 1a+2 (n = 1) 0.35%, 1b+2 (n = 1) 0.03%, and (n = 622) 19.95% patients were not detected. The frequency of genotype 3 was found to be (n = 304) 72.73%, 1a (n = 33) 7.89%, 1b (n = 6) 1.43%, 2 (n = 3) 0.72%, 4 (n = 1) 0.24%, mixed genotype 3+4 (n = 2) 0.48%, 1a+1b+3 (n = 1) 0.24%, and (n = 68) 16.27% patients were not detected in age group > 60 (Figure 2).

Age distribution of HCV genotypes in male patients

**Figure 3:**
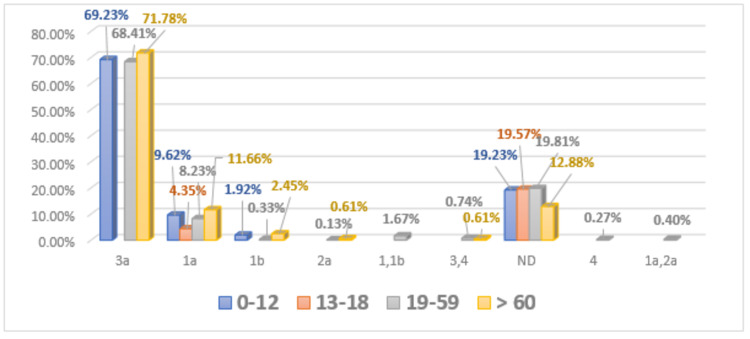
Age-wise distribution of HCV genotypes in the male population HCV, Hepatitis C virus.

The distribution of HCV genotype 3 was (n = 36) 69.23%, 1a (n = 5) 9.62%, and 1b (n = 1) 1.92% was determined in male patients of age group 0-12; (n = 10) 19.23% of patients were found to be not detected in males of age group 0-12. The prevalence of genotype 3 was observed (n = 35) 76%, 1a (n = 2) 4.35%, and (n = 9) 19.57% of patients were not detected in males of age group 13-18. The frequency of genotype 3 had been reported (n = 1022) 68.41%, 1a (n = 123) 8.23%, 1b (n = 5) 0.33%, 2 (n = 2) 0.13%, 4 (n = 4) 0.27%, mixed genotypes like 1+1b (n = 25) 1.67%, 3+4 (n = 11) 0.74%, 1a+2 (n = 6) 0.40% in males of age group 19-59. (n = 296) 19.81% of male patients of age group 19-59 were found to be not detected. In age group > 60, the HCV genotype 3 was detected (n = 117) 71.78%, 1a (n = 19) 11.66%, 1b (n = 4) 2.45%, 2 (n = 1) 0.61%, mixed genotype like 3+4 (n = 1) 0.61%, and (n = 21) 12.88% male patients were not detected (Figure 3).

Age distribution of HCV genotypes in female patients

**Figure 4:**
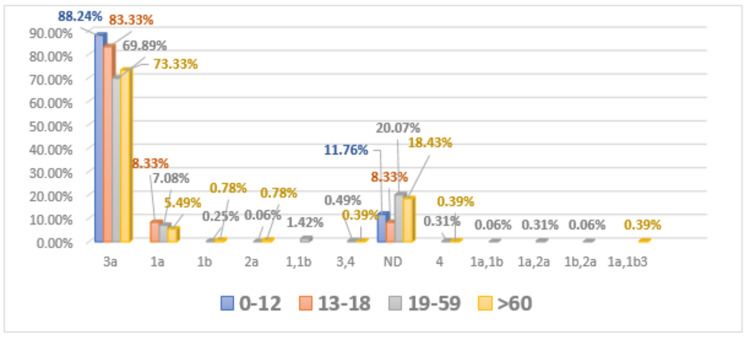
Age-wise distribution of HCV genotypes in the female patients HCV, Hepatitis C virus.

The distribution of HCV genotype 3 was (n = 30) 88.24%, and (n = 4) 11.76% females were found to be not detected in age group 0-12. The frequency of genotype 3 was determined to be (n = 20) 83.33%, 1a (n = 2) 8.33%, and (n = 2) 8.3% patients were not detected in females of age group 13-18. The occurrence of genotype 3 had been reported (n = 1135) 69.89%, 1a (n = 115) 7.08%, 1b (n = 4) 0.25%, 2 (n = 1) 0.06%, 4 (n = 5) 0.31%, mixed genotypes like 1+1b (n = 23) 1.42%, 3+4 (n = 8) 0.49%, 1a+1b (n = 1) 0.06%, 1a+2 (n = 5) 0.31%, and 1b+2 (n = 1) 0.06% in females of age group 19-59. (n = 326) 20.07% of female patients of age group 19-59 were found to be not detected. In age group > 60, the HCV genotype 3 was detected (n = 187) 73.33%, 1a (n = 14) 5.49%, 1b (n = 2) 0.78%, 2 (n = 2) 0.78%, 4 (n = 1) 0.39%, mixed genotype like 1a+1b+3 (n = 1) 0.39%, and (n = 47) 18.43% female patients were not detected (Figure 4).

## Discussion

The prevalence of different HCV genotypes has been assessed through multiple epidemiological studies worldwide. These studies have a role in elucidating the assessment of therapeutics when therapy is prescribed to HCV patients. Multiple clinical trials have helped to resolve epidemiological issues; thus, it has also been shown that the genotype of HCV is associated strongly with different factors like disease severity, prognosis, and response to HCV infection after treatment [15]. As the world is facing an increase in the socioeconomic burden of HCV infection, more reliable information, as well as an understanding of viral pathogenesis, is required to develop new therapeutic strategies. In Pakistan, the regional prevalence of HCV genotypes has shown considerable differences; thus, the knowledge on the diagnosis of HCV genotypic variants is significant for the prognostic indications in chronic HCV patients. In this study, the prevalence of various HCV genotypes in several districts of Punjab was assessed. Data analysis demonstrated that the HCV genotype 3 was more frequent in females (70.8%) than in males (68.9%). These results are in compliance with the outcomes of previous studies conducted in different regions of the country, which also indicated HCV 3 genotype to be the most prevalent genotype reported [16]. Other Asian countries (e.g., India and Nepal) also showed a similar prevalence pattern with a higher frequency of HCV genotype 3 [17], whereas South Asian Countries (e.g., Vietnam, Thailand, and Japan) indicated a different pattern with HCV genotype 1a being the most prevalent genotype [18]. In our data interpretation, the next significantly increasing genotype was 1a (8.4%) in male patients and (6.7%) in female patients. The results in the current study are in conformity with those reported by Haqqi et al. indicating an increase in HCV genotype 1 (a or b) compared to the HCV genotype 3 in Pakistan. Moreover, they also predicted a possible replacement of HCV genotype 3 with genotype 1 (a or b) in the next 10-15 years [19]. Aziz et al., however, has reported a lower frequency of 1a in their study [20].

The management of the upcoming HCV outbreaks may become more complicated because of the proposed shifting pattern of HCV genotypes since the ongoing treatment regimens of genotype 1 demonstrate a weak response [21]. Simultaneously, there is a significant increase in the tendency of having a coinfection of multiple HCV genotypes in the population of Pakistan [22]. In this study, 2.24% patients demonstrated mixed genotype infection with a combination of genotype 3+4 (0.68%) in males and (0.46%) in females, followed by 1a+2 (0.34%) in males and (0.25%) in females, 1a+1b (0.05%) in females, 1b+2 (0.05%) in females, and 1a+1b+3 (0.05%) in female patients.

Davis reported that therapy response of anti-HCV and progression of the disease is influenced by detection of dual subtype in a single patient [23]. Results were analyzed further for the determination of the age-wise distribution of different genotypes of HCV in males and females. The highest distribution was observed in individuals of the 19-59 age group. The results of this study are found in conformity with results reported by Ahmad et al. [24]; they observed a high occurrence of HCV infection in individuals less than 40 years of age, and the same results were reported by Ali et al. [25]. Previously, it has been published that common HCV infection was observed in elder people > 60 years of age group in Pakistan. This situation might be prevailing due to inappropriate identification of HCV infection and lack of awareness seminars in the general population of this region [26].

In the current study, the genderwise frequency pattern of different HCV genotypes was also studied. These results demonstrated no significant variation between the different genotypes of HCV in males and females as all genotypes of HCV were equally distributed between the male and female patients.

Hussain et al. reported no significant difference in concordance with the findings of this study; all the genotypes of HCV recognized were equally distributed in the female and male patients [27]. Results of this study were found in contrast to a study conducted in Luxembourg, in which statistically genotype 3 was more frequent in males, whereas genotype 2 was more prevalent in females [28].

Limitation

There are various limitations that exist in the results of the current study. There is no data available for the correlation of different circulated genotypes to the risk factors associated with possible routes of transmission in our studied population.

## Conclusions

The most frequently distributed genotype of HCV in the examined population of Punjab, Pakistan, was genotype 3, followed by 1a. Equivalent distribution of several HCV genotypes has been seen in males and females of the Punjab province. In addition, the genotype of HCV 1a percentage is equally increasing significantly in both males and females. If this shifting pattern continues to exist, then it may render a challenging situation in terms of antiviral drugs (HCV treatment) and economic burden of the country. The most affected age group was found to be 19-59 years. Likewise, both male and female populations were found to be equally infected with different HCV genotypes. However, this study did not aim at the comparison of different HCV genotypes and the determination of the most frequently distributed genotype in other provinces of Pakistan.
